# The Role of Sleep and the Effects of Sleep Loss on Cognitive, Affective, and Behavioral Processes

**DOI:** 10.7759/cureus.84232

**Published:** 2025-05-16

**Authors:** Anna Hyndych, Rima El-Abassi, Edward C Mader

**Affiliations:** 1 Sleep and Health Research Program, University of Arizona College of Medicine - Tucson, Tucson, USA; 2 Department of Neurology, Louisiana State University Health Sciences Center, New Orleans, USA

**Keywords:** attention, behavior, cognition, daytime performance, emotion, executive function, memory, sleep, sleep deprivation, social

## Abstract

Sleep is essential for various cognitive, affective, and behavioral processes, including attention, memory, executive function, emotional regulation, and interpersonal interactions. Sleep disruptions undermine these functions, resulting in measurable impairments in daily activities, occupational performance, and public safety. Adequate sleep supports sustained vigilance and concentration, whereas sleep deprivation is associated with attentional lapses, diminished cognitive control, and impaired sensory processing. Memory consolidation, which depends on both non-rapid eye movement (NREM) and rapid eye movement (REM) sleep, is particularly susceptible to disruption by sleep loss. Executive functions, such as working memory, impulse control, and decision-making, are notably impaired due to the prefrontal cortex’s heightened sensitivity to insufficient sleep. Sleep deprivation increases amygdala reactivity, weakens prefrontal-amygdala connectivity, and contributes to emotional dysregulation, impulsivity, and risk-taking behaviors. Chronic sleep deprivation exacerbates cognitive deficits, emotional instability, and motor performance decline, leading to higher error rates and reduced workplace productivity. Given its widespread consequences, chronic sleep deprivation constitutes a significant public health concern. This narrative review synthesizes contemporary research on the effects of sleep and sleep loss on waking behavior, with a focus on attention, memory, executive function, emotional regulation, and daytime performance.

## Introduction and background

Sleep is a fundamental biological state, essential for optimal functioning during wakefulness. Human sleep is broadly categorized into two main stages: non-rapid eye movement (NREM) sleep and rapid eye movement (REM) sleep. NREM sleep is characterized by slow-wave brain activity and is critical for restorative processes, memory consolidation, and synaptic plasticity, while REM sleep involves heightened brain activity, REMs, and muscle atonia, and is associated with emotional processing and memory integration [1].

Key physiological processes, including homeostatic regulation, molecular biosynthesis, and neural circuit development, are more effectively executed during sleep [2]. The successful completion of these processes is critical for normal daytime cognition and behavior, including attention, memory, executive function, decision-making, emotional regulation, and social engagement. Disruptions in these processes through sleep deprivation or dysregulation can lead to attentional lapses, mood disturbances, irritability, impaired judgment, strained social interactions, diminished motor skills, and a range of cognitive and behavioral impairments [2].

Measuring the effects of sleep or sleep loss on cognitive function is complex for several reasons: (1) different cognitive domains may rely on sleep processes to varying degrees, (2) circadian rhythms and fluctuations in sleepiness can confound cognitive assessments, (3) individual variability in susceptibility to sleep deprivation exists, and (4) the consequences of sleep deprivation are often domain-specific rather than global [3,4]. Furthermore, much of the current literature has not sufficiently distinguished the roles of NREM sleep from REM sleep, despite growing evidence that these sleep stages serve distinct roles in cognitive processing [5].

Although previous research has extensively examined individual cognitive domains, a comprehensive integration of findings that explicitly links distinct sleep stages and specific cognitive and emotional processes remains limited. Furthermore, current literature often overlooks how sleep disruptions systematically translate into broader, real-world cognitive, emotional, and behavioral consequences.

While earlier studies have primarily emphasized sleep’s impact on attention and memory, emerging research underscores its critical role in emotional processing and behavioral regulation. Sleep deprivation has been linked to emotional dysregulation, heightened impulsivity, and changes in social conduct and risk-taking behavior [3,6-8]. Additionally, excessive daytime sleepiness (EDS) - commonly resulting from insufficient or poor-quality sleep - further exacerbates cognitive dysfunction, undermining an individual’s ability to perform effectively in academic, occupational, and social environments [9-12].

Given the prevalence and significant consequences of sleep disturbances, clarifying the mechanisms by which sleep and sleep deprivation impact cognitive, emotional, and behavioral functions is essential. Understanding the bidirectional relationship between sleep and cognition is crucial, particularly in the context of today's fast-paced, sleep-deprived society, where chronic sleep restriction is increasingly prevalent.

This narrative review examines the impact of sleep and sleep loss on attention, memory, executive function, emotional regulation, and daytime performance. By synthesizing current research, we aim to offer a comprehensive understanding of how sleep promotes cognitive, emotional, and behavioral functioning and how its loss can disrupt these processes through well-defined neurobiological and psychological pathways.

## Review

Attention

 *Sleep and Attention*

Adequate sleep is essential for maintaining attentional control, cognitive vigilance, and sensory integration, ensuring optimal performance in tasks requiring sustained focus [2,13]. During NREM sleep, the brain undergoes critical restorative processes that enhance various cognitive functions, including attention [2,14]. One key mechanism for enhancing attentional performance is the activity of sleep spindles. These neural oscillations play a pivotal role in stabilizing the brain’s attentional networks, with increased spindle activity linked to enhanced attentional efficiency, faster reaction times, and a reduction in lapses of vigilance [15].

Lustenberger et al. found that individuals with higher sleep spindle activity not only demonstrated greater general cognitive ability but also learned verbal declarative material more efficiently [15]. Additionally, experimental studies utilizing the Psychomotor Vigilance Task (PVT) - which measures sustained attention and response speed - have shown that longer total sleep duration and improved sleep efficiency are correlated with faster reaction times and fewer attentional lapses.

Leong et al. conducted a meta-analysis and found that afternoon naps significantly improved performance on attention and memory tasks, particularly in individuals who were sleep-restricted, supporting the restorative effects of sleep on cognitive functioning [16].

Effects of Sleep Loss on Attention

Sustained attention, or vigilance, is one of the cognitive functions most heavily reliant on sleep and, consequently, one of the most vulnerable to sleep deprivation. Vigilance refers to the ability to maintain consistent focus and respond appropriately to stimuli over prolonged periods, a function crucial for a wide range of daily activities, such as driving, learning, and working.

Insufficient sleep significantly impairs vigilance, leading to increased attentional lapses and a decline in both response accuracy and speed [17-19]. Killgore reviewed evidence demonstrating that even moderate sleep deprivation results in slower cognitive reaction times and increased variability in attention and vigilance performance, particularly in tasks requiring sustained focus [17].

Studies employing the PVT consistently demonstrate that well-rested individuals outperform sleep-deprived counterparts on measures of sustained attention. Lim and Dinges conducted a meta-analysis revealing that even short-term sleep deprivation significantly impairs attention and vigilance, with large effect sizes observed for tasks requiring sustained focus [18].

Sleep deprivation disrupts the functioning of key brain regions involved in attention regulation, such as the prefrontal cortex and thalamus, which are responsible for maintaining alertness and attentional focus. Thomas et al. used PET imaging to show that 24 hours of sleep deprivation significantly reduced metabolic activity in these regions, correlating with decreased cognitive performance and alertness [20].

Functional neuroimaging studies have shown that reduced prefrontal cortex activity during task performance is associated with increased variability in reaction times and attentional instability. Chee and Choo demonstrated using functional magnetic resonance imaging (fMRI) that after 24 hours of total sleep deprivation, participants exhibited significantly diminished activation in the dorsolateral prefrontal cortex (DLPFC) during working memory tasks, which was accompanied by slower responses and greater performance variability, underscoring the vulnerability of attentional processes under sleep deprivation [21].

Memory

Sleep and Memory

Memory formation is a dynamic process that involves both wakeful experiences and sleep-dependent consolidation. While synaptic plasticity - the ability of neural connections to strengthen or weaken over time - primarily occurs during wakefulness, sleep plays an essential role in stabilizing, integrating, and enhancing newly acquired information [22,23].

The mechanisms underlying sleep-dependent memory processing involve interactions between the hippocampus and neocortex. Abel et al. outlined that sleep supports long-term memory consolidation through the reactivation and redistribution of hippocampal memory traces to neocortical sites, reinforcing synaptic connectivity involved in recent learning [22]. Additionally, Tononi and Cirelli proposed that slow-wave sleep (SWS) facilitates global synaptic downscaling, which improves signal-to-noise ratios and enhances memory precision by filtering out irrelevant neural activity [23].

The relationship between sleep and memory has been extensively studied, with evidence indicating that both NREM and REM sleep contribute to memory consolidation, albeit through distinct mechanisms. Declarative memory, which encompasses facts and events, is particularly supported by NREM sleep - especially SWS - which promotes hippocampal-neocortical communication essential for memory stabilization. Procedural memory, involving motor skills and learned behaviors, appears to rely more on REM sleep. Genzel et al. emphasized that while individual sleep stages contribute to specific memory types, it is the cyclic alternation between NREM and REM sleep that facilitates optimal memory consolidation by enabling stabilization, integration, and abstraction across complementary processes [24].

Effects of Sleep Loss on Memory

Meta-analyses spanning five decades of research consistently confirm that sleep deprivation impairs both memory encoding and consolidation, underscoring the bidirectional relationship between sleep and memory processes. Total sleep deprivation prior to learning significantly limits the brain’s capacity to encode new information, while sleep loss following learning disrupts consolidation, resulting in increased forgetting of previously acquired material [25,26]. Newbury et al. demonstrated that the detrimental effects of sleep deprivation are evident across diverse populations and experimental designs, with consistent impairments observed in both pre-learning and post-learning conditions [25]. These findings emphasize the critical role of sleep not only in enhancing memory retention but also in preparing the brain for future learning by optimizing neural plasticity and cognitive efficiency.

While the effects of acute total sleep deprivation on memory are well-documented, the long-term consequences of chronic partial sleep restriction remain less well understood. Many individuals accumulate sleep debt through consistent, mild sleep loss, which may subtly but significantly impair memory function over time. Cousins and Fernández reported that repeated partial sleep deprivation can lead to measurable declines in both declarative and procedural memory performance, emphasizing that even subclinical reductions in sleep duration may negatively impact cognitive outcomes [26].

Executive function

Sleep and Executive Function

Executive function encompasses a broad range of higher-order cognitive processes that allow individuals to plan, monitor, and execute goal-directed behavior. These include attentional control, working memory, cognitive flexibility, problem-solving, impulse regulation, and decision-making [27]. The prefrontal cortex, a region critical for executive functions, exhibits dynamic changes in activity during sleep. Dang-Vu et al. highlighted that during normal sleep, functional neuroimaging consistently shows reduced prefrontal cortex activity in slow-wave sleep and increased activation in limbic and paralimbic structures during REM sleep, reflecting the complex role of sleep in regulating brain function [13].

Emerging evidence underscores the significance of both sleep quality and duration in sustaining executive function. Meta-analytic and systematic reviews have shown that good sleep continuity and shorter sleep latency are consistently associated with enhanced executive performance, whereas sleep fragmentation and extended wake after sleep onset are linked to deficits in executive functioning [28-30].

Furthermore, the relationship between sleep duration and executive performance follows a nonlinear pattern. Tai et al. reported that approximately seven hours of sleep per night is optimal for cognitive performance, with both shorter and longer durations associated with impairments in executive abilities [31]. These findings highlight the importance of achieving both adequate and high-quality sleep to support complex cognitive processes.

Effects of Sleep Loss on Executive Function

Acute sleep deprivation, typically defined as one or more nights without sleep, profoundly disrupts executive function. One of its most prominent effects is the impairment of working memory. Functional neuroimaging studies have shown that the DLPFC - a region essential for the temporary storage and manipulation of information - exhibits markedly reduced activation following sleep loss. Chee and Chuah demonstrated that this diminished DLPFC activity compromises the brain's ability to retain task-relevant information, thereby impairing cognitive processes required for planning, reasoning, and goal-directed behavior [32].

Another significant consequence of sleep deprivation is a decline in cognitive flexibility - the capacity to adjust thinking and behavior in response to changing goals or environmental stimuli [28]. Sleep-deprived individuals demonstrate difficulty in modifying their cognitive strategies, frequently exhibiting perseverative errors and a tendency toward rigid, inflexible thinking. Whitney et al. found that total sleep deprivation impairs decision-making that requires adaptive responses to feedback, indicating a blunted capacity to update strategies based on new information [33]. This impairment compromises effective problem-solving and reduces adaptability in dynamic real-world situations.

Initially, the brain attempts to compensate for executive dysfunction by increasing prefrontal cortex activation; however, these compensatory mechanisms become progressively less effective with sustained sleep deprivation [33]. Whitney et al. observed that individuals undergoing total sleep deprivation exhibited impaired feedback-based decision-making, suggesting that despite an initial effort to maintain cognitive performance, executive processes such as adaptive control and strategic thinking begin to deteriorate over time [33]. Notably, individuals may continue to perceive their performance as unimpaired, even in the face of objective deficits, which poses serious risks in environments that demand accurate self-monitoring, risk evaluation, and regulatory control, such as professional, academic, and high-stakes operational settings [33].

Emotion and behavior

Sleep and Emotional Regulation

Sleep plays a critical role in emotional regulation by influencing how individuals process, interpret, and respond to emotional stimuli. The prefrontal cortex is central to this regulation, providing top-down control over the amygdala, which governs emotional reactivity. Adequate sleep strengthens functional connectivity between these regions, promoting balanced emotional responses and resilience to stressors. Yoo et al. demonstrated that sleep deprivation disrupts this prefrontal-amygdala connectivity, resulting in heightened amygdala activation and exaggerated emotional responses to negative stimuli, reflecting diminished regulatory control [6].

REM sleep, in particular, plays a vital role in emotional processing by consolidating emotionally salient memories and modulating the affective intensity of negative experiences. Denis et al. found that REM sleep preferentially strengthens the negative emotional components of memories, which may serve an adaptive role in preparing individuals to respond more effectively to similar future events [34]. This stage facilitates the integration of emotional experiences into broader memory networks, thereby supporting emotional resilience.

NREM sleep also contributes to emotional regulation by dampening amygdala hyperactivity and reducing the emotional charge of stored memories. As Deliens et al. suggest, this process involves the unbinding of core memories from their affective load, which promotes more balanced emotional responses during waking life [35].

Effects of Sleep Loss on Emotional Processing and Behavior

Sleep deprivation profoundly affects emotional regulation, leading to heightened emotional reactivity, reduced impulse control, and increased vulnerability to stress. The prefrontal cortex and amygdala-critical regions involved in modulating emotional responses-are particularly sensitive to the effects of sleep loss. Zaccaro et al. emphasize that insufficient sleep disrupts the functional connectivity between these regions, impairing the prefrontal cortex’s top-down regulation of the amygdala and resulting in exaggerated emotional responses and diminished self-regulation [7].

One of the most notable consequences is emotional volatility: sleep-deprived individuals exhibit increased amygdala activation in response to negative stimuli, accompanied by reduced regulatory input from the prefrontal cortex. This imbalance manifests as irritability, mood swings, and heightened reactivity to minor stressors, contributing to greater susceptibility to anxiety, impulsivity, and poor emotional coping [6].

Sleep deprivation significantly impairs social cognition and increases engagement in risk-related behaviors. One well-documented effect is the disruption of facial emotion recognition - an essential skill for effective communication. Van Egmond et al. found that sleep-deprived individuals struggle to accurately distinguish between positive and negative emotional expressions, contributing to misinterpretations of social cues and increased interpersonal conflict [8]. These deficits in social processing often lead to strained relationships and may contribute to social withdrawal.

Krause et al. demonstrated that chronic sleep deprivation alters the activity of the mesolimbic reward system, heightening impulsivity, risk-seeking behavior, and the preference for immediate gratification [3]. These neural changes help explain the increased incidence of high-risk decisions among sleep-deprived individuals, including poor financial judgment, reckless driving, and substance misuse.

Beyond the acute effects of sleep deprivation, chronic sleep disorders such as primary insomnia also contribute to dysregulation of cognitive and emotional systems. Neuroimaging studies help clarify how sleep-related disturbances impact large-scale brain networks involved in executive functioning and emotional regulation. Wang et al. found that individuals with primary insomnia exhibit altered connectivity between the striatum, default mode network (DMN), and sensorimotor networks, suggesting a reorganization of resting-state activity that may underlie cognitive and affective impairments associated with poor sleep [36]. These findings point to potential neural mechanisms through which disrupted sleep contributes to reduced cognitive control and emotional dysregulation.

Daytime performance

Sleep and Daytime Performance

Sleep is essential for maintaining both cognitive and motor performance, which are critical for effective functioning in professional, academic, and social domains. During sleep, the brain engages in restorative processes that enhance vigilant attention, stabilize reaction times, and support precise motor coordination. As reviewed by Hudson et al., sleep deprivation undermines these functions by impairing the neural mechanisms that sustain alertness and executive control, particularly within the prefrontal cortex and thalamus [19].

REM sleep contributes specifically to adaptive decision-making and creative problem-solving. According to Walker and Stickgold, REM sleep facilitates the reorganization and integration of recently acquired information, allowing individuals to respond more flexibly to complex and novel situations [37].

Sleep's role in daytime performance extends beyond cognitive processes to encompass physical functioning and motor control. It facilitates neuromuscular recovery, supports motor learning, and optimizes reflex responsiveness - all essential for occupations that demand high levels of precision, rapid decision-making, and physical endurance. Grandner highlights that adequate sleep enhances coordination, balance, and psychomotor performance, contributing to greater productivity and reduced error rates across various occupational and academic domains [38]. In contrast, insufficient sleep compromises these functions, increasing the risk of accidents and reducing overall efficiency in daily activities.

Effects of Sleep Loss on Cognitive and Motor Performance

Both acute (one night of total sleep loss) and chronic (persistent sleep restriction) sleep deprivation significantly impair cognitive and motor function, leading to increased errors, reduced productivity, and a heightened risk of accidents. One of the immediate effects of sleep loss is a decline in vigilant attention, which is essential for detecting and responding to critical stimuli.

Studies show that sleep-deprived individuals exhibit slower response times, increased attentional lapses, and greater variability in task performance, making them more prone to mistakes and accidents, particularly in high-stakes environments. Hudson et al. found that sleep deprivation compromises sustained attention by disrupting functional connectivity between the prefrontal cortex and thalamus, with neuroimaging studies revealing changes in activation patterns in these regions after sleep loss [19].

Cognitive deficits from sleep deprivation extend beyond impairments in vigilance and reaction time, encompassing disruptions in impulse control, emotional regulation, and decision-making. According to Killgore and Weber, sleep deprivation compromises prefrontal cortex function, which plays a crucial role in modulating executive processes, including inhibitory control and risk evaluation. As a result, individuals experiencing sleep loss exhibit increased emotional reactivity, reduced stress tolerance, and impaired judgment [39]. These disruptions in executive function impair inhibitory control and risk assessment, contributing to increased emotional reactivity, workplace conflicts, and impulsive decision-making.

Lowe et al. conducted a meta-analysis showing that even moderate sleep restriction results in significant impairments in attention, working memory, and higher-order cognitive functions associated with the prefrontal cortex, reinforcing the critical role of sleep in maintaining executive control [40].

Motor performance is similarly impaired by sleep deprivation. Hudson et al. described how sleep loss alters brain function in regions responsible for motor control, such as the motor cortex and cerebellum, contributing to slower reaction times and increased variability in motor output [19]. These deficits manifest as poor coordination and decreased fine motor skills, which are especially concerning in high-risk professions like healthcare, aviation, and law enforcement, where split-second decisions are crucial to safety outcomes. Grandner further emphasized that cumulative sleep debt is associated with deteriorations in psychomotor performance and physical stamina, increasing the risk of workplace injuries and reduced productivity over time [38].

The role of sleep and the effects of sleep loss on attention, memory, executive function, emotions and behavior, and daytime performance are summarized in Table 1.

**Table 1:** Summary of the role of sleep and the effects of sleep loss on cognitive function and behavioral regulation. Table Credits: Edward C. Mader, Jr.

Cognition and behavior	Role of normal sleep	Brain structures involved	Effects of sleep loss
Attention	Enhances sustained attention and vigilance, supports selective attention	Prefrontal cortex, parietal lobes, thalamus	Reduced alertness, slower reaction times, increased distractibility, impaired sustained attention
Memory	Facilitates consolidation of declarative and procedural memory, strengthens neural connections	Hippocampus, neocortex, medial temporal lobe	Impaired memory consolidation, difficulty in learning new information, increased forgetfulness
Executive function	Supports problem-solving, decision-making, cognitive flexibility, and impulse control	Prefrontal cortex, anterior cingulate cortex, basal ganglia	Poor decision-making, reduced cognitive flexibility, impulsivity, impaired problem-solving
Emotion and behavior	Regulates emotional reactivity and mood stability	Amygdala, prefrontal cortex, limbic system	Increased emotional reactivity, mood instability, heightened stress response, risk-taking behavior
Daytime performance	Maintains cognitive efficiency, motor coordination, and overall functional performance	Prefrontal cortex, cerebellum, basal ganglia	Reduced work efficiency, impaired motor coordination, increased risk of accidents, microsleeps

Sleepiness: a confounding factor in sleep-behavior research

Sleepiness: Normal and Abnormal Physiology

Sleepiness is a distinct physiological state that fluctuates throughout the day, influenced by circadian rhythms and homeostatic sleep pressure. It is not synonymous with sleep or sleep deprivation, as it reflects an individual’s propensity to transition into sleep rather than the absence of sleep. Under typical conditions, sleepiness peaks at night in alignment with the circadian drive and experiences a mild increase in the mid-afternoon, corresponding to a natural dip in alertness [9]. Carskadon and Dement found that nocturnal sleep quality, timing, and continuity are key nocturnal determinants of next-day sleepiness, demonstrating that disruptions in sleep architecture significantly influence daytime alertness [9]. This cyclical variation in sleepiness is governed by the interactions between the suprachiasmatic nucleus and homeostatic sleep mechanisms, which ensure stable wakefulness and facilitate sleep-wake transitions [41].

EDS, by contrast, represents a pathological deviation from normal sleep-wake physiology, characterized by an increased tendency to fall asleep inappropriately during the day [42]. It is commonly quantified using the Epworth Sleepiness Scale (ESS), a widely employed subjective tool for assessing sleepiness [42].

EDS is linked to dysfunction in wake-promoting systems, including alterations in the prefrontal cortex, thalamus, and brainstem arousal pathways [41]. Saper et al. described the hypothalamus as a critical integrator of circadian and homeostatic signals, identifying distinct populations of neurons in the brainstem and hypothalamus - including orexinergic and histaminergic neurons - that play essential roles in stabilizing wakefulness and maintaining arousal [41].

The orexin/hypocretin system, which plays a critical role in regulating wakefulness, is particularly implicated in disorders associated with sleepiness, such as narcolepsy [43]. Scammell et al. highlighted that orexin neurons, located in the lateral hypothalamus, promote arousal by activating monoaminergic and cholinergic neurons, and their dysfunction leads to instability in wake-sleep transitions, as seen in narcolepsy with cataplexy [43].

Structural neuroimaging has also demonstrated that reduced gray matter volume in regions such as the ventromedial prefrontal and orbitofrontal cortex correlates with elevated ESS scores. Killgore et al. found significant negative correlations between subjective daytime sleepiness and gray matter volume in these frontal regions, suggesting that decreased structural integrity in areas critical for arousal and executive control may underlie excessive sleepiness [10].

While minor variations in daytime sleepiness are typical within the sleep-wake cycle, persistent or excessive sleepiness often indicates underlying dysfunctions in sleep regulation, circadian alignment, or arousal mechanisms [43]. This underscores the need to distinguish between normal physiological sleepiness and pathological conditions contributing to EDS, particularly in clinical and occupational settings where sustained alertness is paramount.

Experimental Evidence: The Impact of Sleepiness on Cognition and Behavior

Experimental research has established that sleepiness exerts profound effects on cognitive and behavioral performance, independent of total sleep loss. Studies show that individuals with high subjective sleepiness scores exhibit impairments in sustained attention, working memory, and reaction time tasks, even when they have received an adequate amount of sleep [12]. Doran et al. demonstrated that lapses in attention during sleep deprivation occur in a highly variable, unstable pattern - termed “state instability” - which reflects fluctuating levels of alertness and suggests that sleepiness and vigilance impairments are not solely dependent on prior sleep duration [11].

Increased frontal theta activity has been identified as a neurophysiological marker of EDS, indicating altered frontal cortical function and diminished large-scale connectivity. Hitchcott et al. found that these changes reflect a shift toward an altered state of consciousness, characterized by impaired cognitive stability, attentional lapses, and reduced awareness [12]. In addition to attentional deficits, individuals with EDS exhibit broader impairments in sensory integration and behavioral responsiveness, supporting the notion that excessive sleepiness represents not merely reduced wakefulness but a distinct neurocognitive state with diminished executive control [12].

These findings highlight the necessity of treating sleepiness as an independent variable in sleep-behavior research, rather than solely attributing cognitive and behavioral impairments to sleep deprivation. Future studies should aim to delineate the mechanisms underlying sleepiness-related deficits, particularly in populations affected by chronic EDS, shift work disorders, and neurological conditions that disrupt wake regulation.

Real-world and clinical implications

The consequences of sleep disturbances extend well beyond the laboratory, significantly influencing academic performance, workplace efficiency, mental health, interpersonal relationships, and public safety. Sleep loss is associated with errors in decision-making, heightened emotional reactivity, increased impulsivity, and diminished cognitive resilience, all of which contribute to occupational hazards, academic underachievement, and a greater risk of psychiatric and neurodegenerative disorders. This section consolidates the real-world and clinical implications of sleep disturbances across cognitive and behavioral domains and explores potential interventions to mitigate their effects.

Real-World Consequences of Sleep Disturbances

Cognitive impairments and workplace performance: Sleep deprivation leads to significant reductions in attentional capacity, working memory efficiency, and executive function, increasing the likelihood of errors, accidents, and decreased productivity. In high-risk professions such as healthcare, law enforcement, transportation, and aviation, even modest reductions in sleep quality or duration can lead to life-threatening mistakes. Lockley et al. reported that extended work hours and insufficient sleep among healthcare providers were associated with increased medical errors and adverse events, particularly in interns working shifts exceeding 24 hours [44]. Similarly, Goel et al. described that sleep deprivation leads to measurable declines in cognitive domains critical to occupational performance, including attention, working memory, and decision-making, with neuroimaging evidence showing altered activation patterns in the prefrontal and parietal cortices [45].

Chronic sleep loss in shift workers and individuals with rotating schedules is associated with elevated workplace fatigue, absenteeism, and reduced job performance. Grandner emphasized that insufficient sleep in occupational settings is linked to increased safety incidents, reduced productivity, and greater healthcare utilization, especially among shift workers who experience circadian misalignment and chronic sleep restriction [38].

Furthermore, sleep-deprived individuals exhibit a diminished ability to assess risks effectively, often underestimating long-term consequences in favor of immediate rewards. Krause et al. demonstrated that sleep deprivation disrupts functional connectivity between the prefrontal cortex and striatal reward systems, leading to impaired risk evaluation and exaggerated reward sensitivity - mechanisms that may underlie suboptimal decision-making in financial, legal, and business contexts [3].

Sleep and academic performance: A robust body of research has demonstrated that sleep plays a critical role in learning efficiency, with sleep deprivation negatively impacting memory encoding, consolidation, and retrieval [14]. Diekelmann and Born emphasized that sleep supports memory consolidation by facilitating neural reactivation and the redistribution of hippocampal memory traces to neocortical areas, enhancing long-term retention [14]. Van der Heijden et al. found that chronic sleep restriction in higher education students was significantly associated with lower academic achievement, impaired problem-solving abilities, and reduced concentration, making it more difficult to retain and apply new information [46].

Adequate post-learning sleep has been shown to enhance recall and retention, while sleep deprivation results in significant deficits in long-term memory storage and cognitive flexibility. Payne et al. demonstrated that participants who slept after learning semantically related word pairs showed better recall compared to those who remained awake, highlighting the role of sleep in strengthening associative memory links [47]. These impairments hinder the brain's ability to integrate new knowledge effectively, complicating academic success.

Additionally, sleep disturbances in students are linked to increased stress, difficulty managing academic tasks, and a greater likelihood of engaging in maladaptive study behaviors, such as procrastination and excessive study habits, ultimately impacting academic performance. Alshammari et al. found that insomnia correlates positively with increased bedtime procrastination and inefficient study behaviors, which exacerbate academic strain and reduce productivity [48]. Interventions aimed at improving sleep hygiene and time management may help enhance academic efficiency and alleviate stress-related disruptions.

Clinical Conditions Impacted by Sleep Disturbances

Sleep dysfunction is a well-established factor in several neurodevelopmental and psychiatric disorders, contributing to attention deficits, emotional instability, impaired cognitive function, and increased risk-taking behavior.

Attention-deficit/hyperactivity disorder (ADHD): Sleep deprivation exacerbates attentional impairments in individuals with ADHD, intensifying symptoms such as inattention, impulsivity, and cognitive dysregulation. Hvolby et al. reported that children with ADHD commonly experience disrupted sleep patterns, including delayed sleep onset and reduced sleep duration, which are closely linked to worsening core ADHD symptoms [49]. Larsson et al. found that behavioral sleep interventions, such as parent-delivered strategies and cognitive-behavioral therapy for insomnia, produced modest improvements in sleep parameters and were associated with enhanced attentional performance in children with ADHD [50].

Depression and anxiety: Sleep disturbances are a core feature of mood disorders, with fragmented sleep contributing to deficits in sustained attention, increased emotional reactivity, and heightened susceptibility to stress-related cognitive impairments. Tomaso et al. conducted three meta-analyses and found that both total and partial sleep deprivation significantly increased negative mood and emotional reactivity, while reducing emotional regulation capacity, supporting the link between inadequate REM sleep and affective dysregulation [51].

Cognitive aging and neurodegeneration: Poor sleep quality further exacerbates age-related declines in attentional control and executive function in older adults. Mander et al. reported that reductions in slow-wave sleep in aging are associated with impaired memory consolidation due to disrupted hippocampal-neocortical communication, contributing to age-related cognitive decline [52]. Scullin and Bliwise summarized five decades of research, concluding that sleep fragmentation and diminished sleep quality in older adults correlate with increased distractibility, memory deficits, and accelerated neurocognitive aging [53].

Potential Interventions for Mitigating Sleep-Related Impairments

Behavioral and cognitive interventions: Cognitive Behavioral Therapy for Insomnia (CBT-I) is an evidence-based treatment that significantly improves sleep quality, reduces sleep latency, and enhances cognitive and emotional functioning. Harvey et al. found that CBT-I outperformed behavior therapy and cognitive therapy alone in reducing insomnia severity and improving overall psychological well-being in patients with chronic insomnia [54]. In addition, Baranwal et al. emphasized that foundational sleep hygiene practices-such as maintaining consistent sleep schedules, reducing stimulant intake before bedtime, and optimizing sleep environments-can improve memory, attention, and impulse regulation, particularly in populations with high cognitive demands [55].

Circadian-based and pharmacological interventions: Bright light therapy and melatonin supplementation are widely used to address circadian rhythm disturbances and associated cognitive and mood impairments. Ferracioli-Oda et al. conducted a meta-analysis of randomized controlled trials and found that melatonin significantly reduced sleep onset latency and increased total sleep time, supporting its utility as a first-line treatment for circadian misalignment and insomnia-related complaints [56]. In individuals with delayed sleep-wake phase disorder and comorbid ADHD, Fargason et al. demonstrated that morning exposure to bright light effectively advanced circadian phase and significantly improved attention and sleep regulation [57].

Pharmacological interventions, particularly stimulant medications such as modafinil and armodafinil, have also been investigated for their role in mitigating cognitive deficits linked to excessive sleepiness. Minzenberg and Carter reviewed that modafinil enhances cognitive performance by modulating catecholaminergic and glutamatergic systems, with observed benefits in executive function, working memory, and attention in populations with narcolepsy, ADHD, and shift work disorder [58]. Sodium oxybate, and more recently, its extended-release formulation, has been shown to improve sleep continuity and reduce EDS in narcolepsy patients, with sustained cognitive and functional benefits in randomized controlled trials [59,60].

Workplace and academic policy adjustments: Fatigue risk management strategies are critical in enhancing performance and safety, particularly in high-risk occupations. Sadeghniiat-Haghighi and Yazdi emphasized that interventions such as regulated work hours, structured rest periods, and designated workplace napping significantly reduce fatigue-related errors and improve overall alertness and productivity [61]. In academic settings, delaying school start times, implementing sleep hygiene education, and promoting cognitive resilience have been shown to support student well-being. Wheaton et al. reviewed that later school start times are associated with increased sleep duration, improved academic performance, and better mental health outcomes among adolescents [62].

Future directions

Despite considerable progress in understanding the cognitive, emotional, and behavioral consequences of sleep deprivation, several important questions remain unresolved. First, longitudinal studies are needed to investigate the cumulative effects of chronic sleep restriction over the lifespan, particularly on neuroplasticity, emotional resilience, and neurodegenerative risk. This is especially relevant for vulnerable populations, including adolescents, shift workers, and older adults, whose sleep architecture may be uniquely impacted by social and biological factors.

Second, future research should aim to disentangle the differential roles of NREM and REM sleep in distinct cognitive domains. Advanced neuroimaging and electrophysiological techniques may help clarify how sleep stages interact to support memory consolidation, attentional control, and executive functioning.

Third, EDS requires greater empirical attention as a standalone contributor to cognitive impairment. Studies that isolate EDS from total sleep duration could improve our understanding of the mechanisms through which momentary lapses in wakefulness degrade performance.

Lastly, intervention-based research should explore the efficacy of personalized, multimodal approaches - including behavioral therapy, pharmacologic agents, and circadian-alignment strategies - to mitigate the adverse effects of sleep disturbances. Translational research linking laboratory findings with real-world functional outcomes will be essential in developing evidence-based policy recommendations and clinical guidelines.

## Conclusions

Sleep is critical for maintaining optimal cognitive function, emotional stability, and overall behavioral performance. Adequate sleep is essential for attentional control, memory consolidation, executive function, and emotional regulation, while sleep deprivation impairs these processes, negatively affecting workplace safety, academic success, and general well-being. The wide-ranging and cumulative consequences of insufficient sleep highlight the importance of promoting sleep health through clinical, occupational, and public health interventions to improve cognitive resilience and quality of life.

Future research should focus on understanding the long-term effects of chronic sleep restriction and developing targeted strategies to mitigate its adverse outcomes. By increasing awareness and implementing evidence-based approaches to manage sleep deprivation, we can foster healthier cognitive and emotional functioning across various populations.
